# Isoform-Specific Contributions of α-Actinin to Glioma Cell Mechanobiology

**DOI:** 10.1371/journal.pone.0008427

**Published:** 2009-12-23

**Authors:** Shamik Sen, Meimei Dong, Sanjay Kumar

**Affiliations:** 1 Department of Bioengineering, University of California, Berkeley, California, United States of America; 2 Institute of Molecular and Cell Biology, Mannheim University of Applied Sciences, Mannheim, Germany; Dalhousie University, Canada

## Abstract

Glioblastoma Multiforme (GBM) is a malignant astrocytic tumor associated with low survival rates because of aggressive infiltration of tumor cells into the brain parenchyma. Expression of the actin binding protein α-actinin has been strongly correlated with the invasive phenotype of GBM in vivo. To probe the cellular basis of this correlation, we have suppressed expression of the nonmuscle isoforms α-actinin-1 and α-actinin-4 and examined the contribution of each isoform to the structure, mechanics, and motility of human glioma tumor cells in culture. While subcellular localization of each isoform is distinct, suppression of either isoform yields a phenotype that includes dramatically reduced motility, compensatory upregulation and redistribution of vinculin, reduced cortical elasticity, and reduced ability to adapt to changes in the elasticity of the extracellular matrix (ECM). Mechanistic studies reveal a relationship between α-actinin and non-muscle myosin II in which depletion of either α-actinin isoform reduces myosin expression and maximal cell-ECM tractional forces. Our results demonstrate that both α-actinin-1 and α-actinin-4 make critical and distinct contributions to cytoskeletal organization, rigidity-sensing, and motility of glioma cells, thereby yielding mechanistic insight into the observed correlation between α-actinin expression and GBM invasiveness in vivo.

## Introduction

Glioblastoma Multiforme (GBM) is a high-grade astrocytoma characterized by aggressive invasion of individual tumor cells into the brain parenchyma [1]. The diffuse infiltration of GBM tumors along vasculature and white matter tracts in the central nervous system makes complete resection virtually impossible, giving rise to a mean survival time from diagnosis of only 1–2 years, even with aggressive therapy. The remarkable invasiveness of GBM tumors is attributed in part to the capacity of the constituent tumor cells to remodel the extracellular matrix (ECM), which is made possible by integrin upregulation [2], matrix metalloprotease (MMP)-mediated proteolysis [3], and de novo secretion of ECM proteins [4]. This remodeling also depends on the ability of the tumor cells to generate actomyosin-based contractile forces, which have been observed in other systems to facilitate ECM fibril remodeling during migration, thereby providing contact guidance cues to invasive cells [5]. The importance of non-muscle myosin II (NMMII) in glioma invasiveness has been demonstrated by studies where inhibition of myosin light chain kinase (MLCK) completely abrogated glioma motility [6].

To explore potential connections between ECM-encoded signals, cellular contractility, and tumor progression, we recently investigated the role of ECM rigidity (stiffness) in controlling behaviors of glioma cells relevant to growth and spread [7]. We demonstrated that the adhesion and cytoarchitecture of a variety of glioma cell culture models are indeed sensitive to ECM stiffness, and that this microenvironmental parameter can profoundly influence cell motility and proliferation. Moreover, NMMII is specifically required for this rigidity sensitivity, with inhibition of NMMII abrogating stiffness-dependent differences in adhesion and rescuing cell motility on highly compliant ECMs. While this study clearly established a connection between ECM-based mechanical cues, tumor cell adhesion and migration, and NMMII activity, the underlying molecular mechanisms remain incompletely understood.

Focal adhesions (FAs) play a central role in transducing mechanical signals between the cytoskeleton and ECM [8]. In the case of GBM specifically, a comparative study of in vitro migration and invasion across ten human GBM cell lines revealed that levels of the FA and actin-binding protein α-actinin correlates directly with biological aggressiveness [9]. The field's understanding of α-actinin function in this context has been complicated by the relatively recent discovery that four distinct isoforms exist in humans: the nonmuscle isoforms 1 and 4 and the muscle-specific isoforms 2 and 3. While both α-actinin-1 and α-actinin-4 have been reported to localize along stress fibers [10], α-actinin-1 also localizes to FAs and cell-cell contacts [11], and α-actinin-4 is also enriched at the leading edges of invading cells [12]. Further, immunohistochemical analysis of human tumors demonstrates that the cytoplasmic localization of α-actinin-4 accurately predicts an infiltrative phenotype and poor clinical prognosis [13], [14], [15].

While the above studies clearly establish that both α-actinin-1 and -4 contribute to tumor invasion and metastasis, the importance of each isoform to underlying cellular mechanobiological properties remains unclear. For example, siRNA-mediated knockdown of α-actinin-1 increases motility and tumorigenicity of fibroblasts, consistent with a role in stabilizing cell-ECM adhesive contacts [16], [17]. In intestinal epithelial cells, suppression of α-actinin-1, but not α-actinin-4, inhibits deformation-induced ERK phosphorylation and proliferation [18], reflecting the role of α-actinin-1 in linking the cytoskeleton to the extracellular matrix (ECM). In ovarian carcinoma cells, α-actinin-4 knockdown leads to reduced motility and invasion [13], whereas mice genetically deficient in α-actinin-4 exhibit *increased* lymphocyte chemotaxis [19]. This heterogeneity of findings illustrates two broader points: First, the role of each isoform is highly cell-type specific, making it difficult to extrapolate these studies to human glioma cells. Second, particularly if α-actinin is to be pursued as a drug target, the field could benefit from additional quantitative and molecular-scale insight into how each isoform contributes to biophysical interactions between tumor cells and the ECM, including adhesion, contractility, and mechanotransduction.

Here we seek to address both of these gaps in our understanding of α-actinin function by investigating the contributions of α-actinin-1 and α-actinin-4 to the structure, mechanics, and motility of human glioma cells. We find that siRNA-mediated suppression of either isoform reduces random cell migration speed and alters the expression and localization of the FA protein vinculin. Strikingly, suppression of either α-actinin isoform reduces ECM rigidity sensitivity and alters responses to pharmacologic inhibition of MLCK and Rho-associated kinase (ROCK). We attribute these differences to alterations in NMMII expression and modulation of contractility through interactions between MLCK and individual α-actinin isoforms. Taken together, our results demonstrate that both α-actinin-1 and α-actinin-4 make critical and distinct contributions to cytoskeletal organization, rigidity-sensing, and motility of glioma cells.

## Materials and Methods

### Cell Culture and RNA Interference

U373-MG human glioma cells were obtained and cultured as previously described [7]. Cells were plated either on glass coverslips or tissue culture polystyrene coated with bovine collagen I (Inamed Biosciences) or on collagen-coated polyacrylamide (PA) hydrogels attached to glass coverslips (see below). To suppress α-actinin expression, cells were transfected with isoform-specific α-actinin siRNA sequences (siACTN1 or siACTN4) or non-targeting sequences (siCTL) (Santa Cruz, CA) per manufacturer's specifications. For MLCK and ROCK inhibition studies, cells were incubated with either ML7 (Calbiochem), Y27632 (Calbiochem) or blebbistatin (Sigma) at 10 µM final concentration. For cell motility experiments, cells were incubated with drug for 1 hour prior to acquisition of time-lapse images.

### Immunofluorescence and Western Blots

Immunofluorescence [7], [20] utilized previously validated primary (mouse anti-α-actinin-1 (1∶200, Santa Cruz); mouse anti-α-actinin-4 (1∶250, Abcam); mouse anti-vinculin (1∶200, Sigma)) and secondary antibodies (Alexa Fluor 543 goat anti-mouse IgG; Alexa Fluor 543 donkey-anti-goat IgG (1∶500, Invitrogen)). F-actin and nuclear stains utilized Alexa Fluor 488-phalloidin (1∶200, Invitrogen) and DAPI (1∶500, Invitrogen), respectively. Immunoblots followed manufacturer specifications (Invitrogen Western Blot kit), using previously validated primary antibodies ((α-actinin-1 (1∶500, Santa Cruz); α-actinin-4 (1∶500, Santa Cruz); NMMII (1∶500, Santa Cruz); vinculin (1∶500, Sigma); phosphorylated MLC (pMLC) (1∶500, Cell Signaling); pY397FAK (1∶500, Invitrogen); GAPDH (1∶20000, Sigma)) and HRP-conjugated secondary antibodies (goat-anti-mouse, donkey-anti-goat or goat-anti-rabbit (Invitrogen)), followed by TMB chromogenic substrate (Invitrogen). For pMLC blots, chemiluminescent rather than colorimetric detection was performed according to manufacturer protocols. After development and scanning, band intensities were quantified using ImageJ (NIH).

### Image Acquisition and Analysis

Imaging was performed using a Nikon TE2000E2 microscope equipped with an incubator chamber for controlled temperature, humidity, and CO_2_. Images were recorded with a CCD camera (Photometrics CoolSNAP HQ2) interfaced to image acquisition software (Compix SimplePCI). Cell spreading areas were measured using ImageJ (NIH) by manually tracing the projected outlines of 50–100 cells per condition. Confocal images were obtained with a Zeiss LSM510 microscope. For quantifying size distribution of FAs, confocal images of vinculin stained cells (10 cells per condition) were thresholded (ImageJ) and quantified for number and size of FAs. FA circularity, defined as 4π * (area/perimeter^2^), ranges between 0 and 1, with a value close to 0 indicating an oblong morphology and a value of 1 indicating a perfectly circular morphology. For motility measurements, cells were imaged every 15 minutes at 10× magnification for >6 hours and quantified using manual tracking in ImageJ. The data was further processed to obtain mean speed for a given condition.

### Synthesis of Polyacrylamide ECMs and Traction Force Microscopy (TFM)

Polyacrylamide ECMs of defined stiffness were synthesized by polymerizing and crosslinking predetermined ratios of acrylamide and bisacrylamide [7], [21]. For TFM experiments [22], [23], Texas red-labeled microspheres (1 µm diameter, Invitrogen) were included in the acrylamide/bisacrylamide solution prior to polymerization. Gels were then covalently decorated with full-length bovine collagen I at a fixed density using the photoactivatable crosslinker sulfo-SANPAH. For TFM, maps of substrate displacement and strain were computed from bead positions before and after cell detachment using Fourier transform traction cytometry [22].

### Atomic Force Microscopy (AFM)

The indentational elasticity of cells cultured on either collagen-coated PA hydrogels or glass coverslips were measured with an Asylum MFP 3-D AFM (Asylum Research, CA) as described previously [20].

## Results

### Localization and Suppression of α-Actinin Isoforms in Cultured Glioma Cells

**Figure 1:**
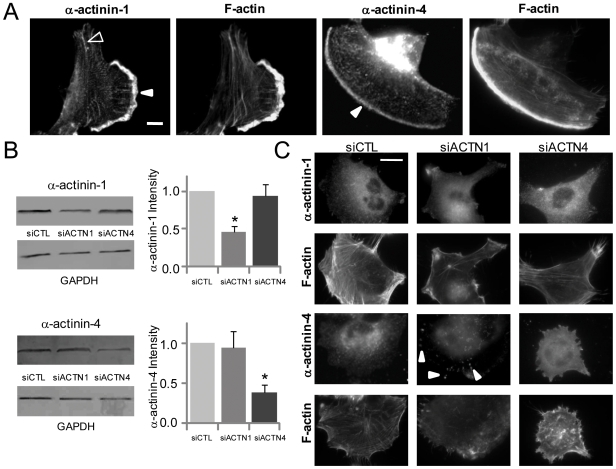
Expression, localization, and suppression of α-actinin isoforms in glioma cells. (A) Localization of α-actinin-1 and α-actinin-4 in U-373 MG cells cultured on collagen-coated glass. Solid and open arrowheads mark the localization of individual α-actinin isoforms at ruffles and along actin stress fibers, respectively. Scale Bar = 10 µm. (B) Measurement of siRNA efficacy by Western Blot of U-373 MG cells transfected with either scrambled control siRNA (siCTL) or siRNA directed against α-actinin-1 (siACTN1) or α-actinin-4 (siACTN4) (*p<0.05). (C) Subcellular localization of α-actinin-1 and α-actinin-4 following siRNA treatment. Arrowheads depict the redistribution of α-actinin-4 to FAs when α-actinin-1 is suppressed. Scale Bar = 20 µm.

To establish baseline expression and localization of α-actinin isoforms in a human glioma cell line we had previously demonstrated to be mechanosensitive [7], we cultured U-373 MG human glioma cells on collagen I-coated glass coverslips and examined isoform distributions by immunofluorescence. While U-373 MG glioma cells were found to exhibit a wide heterogeneity in cell morphology, consistent with our and others' previous reports, membrane ruffles were observed in ∼48% of cells. In this population of cells, both α-actinin-1 and α-actinin-4 localized strongly to membrane ruffles (Figure 1A). In addition, α-actinin-1 stained in a focal adhesion-like distribution in ∼70% of cells and colocalized with stress fibers in ∼35% of cells. In comparison to α-actinin-1, α-actinin-4 localized along stress fibers in ∼45% of cells. We next suppressed expression of each isoform with isoform-specific siRNA sequences (siACTN1 and siACTN4 respectively) and compared the results of each treatment on expression and subcellular localization to each other and to a scrambled siRNA control sequence (siCTL) (Figure 1B). Individual isoform-specific siRNA sequences and transfection conditions were optimized to obtain ∼55% depletion of α-actinin-1 and ∼60% depletion of α-actinin-4 by immunoblot, comparable to levels of suppression achieved in previous studies with minimal off-target effects [18]. Consistent with this incomplete knockdown, α-actinin-1 and α-actinin-4- depleted cells still stained weakly positive for α-actinin-1 and α-actinin-4 respectively (Figure 1C, Figure S1). Although depletion of one isoform did not cause compensatory changes in expression of the other isoform, α-actinin-1 suppression led to partial redistribution of α-actinin-4 to FAs (arrows). This was also confirmed by co-staining cells with isoform-specific α-actinin antibodies and an antibody against the phosphorylated Tyrosine-397 of focal adhesion kinase (pY397FAK), a marker of mature focal adhesions (Figure S1).

### α-Actinin Isoforms Contribute to Glioma Cell Motility

**Figure 2:**
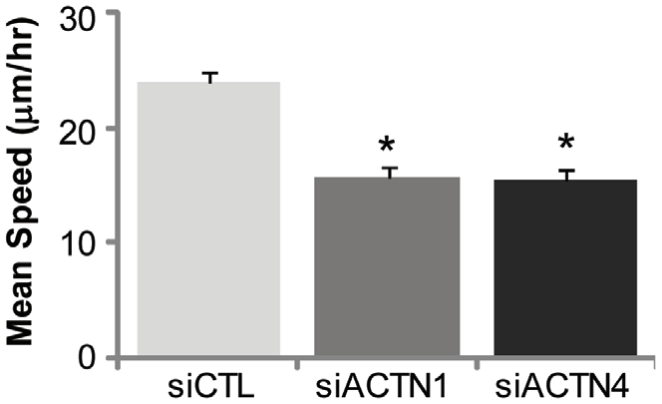
Contributions of α-actinin isoforms to glioma cell motility. Effect of α-actinin depletion on mean speeds (mean±SEM, *p<0.001) of cells tracked over 6 hours.

We next quantified the effect of depleting each α-actinin isoform on cell motility *in vitro* by tracking the random migration of control and knockdown cells using phase-contrast time-lapse imaging (Figure 2, Movies S1, S2, S3). The contributions of individual α-actinin isoforms to glioma motility were quantified by calculating and comparing mean migration speeds for each condition (Figure 2). We found that knockdown of either isoform caused a ∼35% reduction in mean cell speed relative to control, indicating that both α-actinin-1 and α-actinin-4 contribute to glioma cell motility.

### α-Actinin Isoforms Contribute to ECM Rigidity Sensing by Glioma Cells

**Figure 3:**
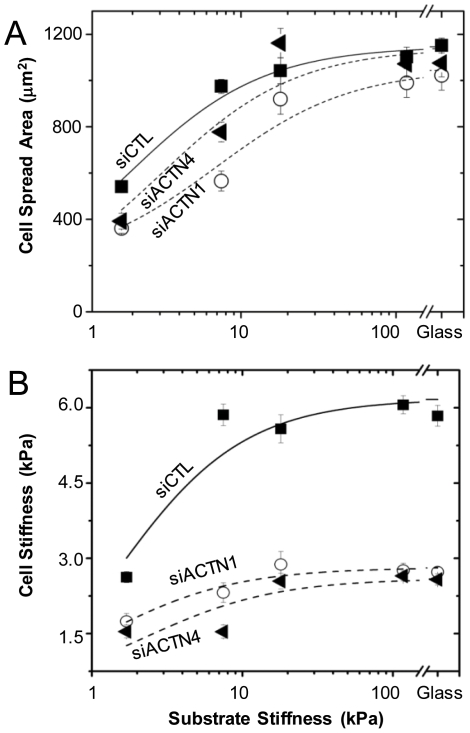
Contributions of α-actinin isoforms to cell-ECM rigidity sensing. (A) Projected cell-ECM adhesion area of siCTL (squares), siACTN1 (circles), and siACTN4 (triangles) cells on collagen I-coated polyacrylamide ECMs of varying elasticity. Cell spreading differences between control and α-actinin depleted cells are statistically significant (p<0.001) for both isoforms on 2 kPa and 8 kPa ECMs. (B) Cortical cell elasticity of siCTL, siACTN1, and siACTN4 cells on variable-rigidity ECMs by AFM. Differences in cortical elasticity between control and α-actinin-depleted cells are statistically significant (p<0.001) for all ECM stiffness.

To test the hypothesis that α-actinin-1 and α-actinin-4 participate in sensing mechanical cues from the ECM and regulating the shape plasticity of glioma tumor cells, we cultured control, α-actinin-1-depleted, and α-actinin-4-depleted cells on a series of collagen I-coated polyacrylamide ECMs spanning a range of elasticities and compared the dependence of projected cell spreading area and cortical stiffness on ECM elasticity (Figure 3). Similar to other cell systems [21], [24], [25], control cells spread poorly on relatively compliant ECMs (<5 kPa), more extensively on stiffer ECMs (8–20 kPa), and to a maximal area above a threshold ECM stiffness (>20 kPa). (Figure 3A). In contrast, both α-actinin-1 and α-actinin-4 depleted cells spread less extensively than controls at low ECM stiffness, with up to ∼25% reduction in spreading area on the most compliant ECMs. On intermediate-stiffness (∼8 kPa) gels, both α-actinin-1 and α-actinin-4 depleted cells were less spread compared to controls, with α-actinin-4 depleted cells spreading ∼40% more than α-actinin-1 depleted cells. On the stiffest gels and on glass, both α-actinin-1 and α-actinin-4-depleted cells achieved spreading areas comparable to control cells.

Consistent with the increase in spreading area from soft (∼2 kPa) gels to intermediate-compliance (∼8 kPa) gels, the cortical stiffness of control cells increased from ∼2.6 kPa on the soft gels to ∼6 kPa on 8 kPa gels, without any further increases in cortical stiffness with increasing ECM stiffness (Figure 3B). This adaptation of cortical stiffness to ECM stiffness has been observed previously in both cultured fibroblasts [26] and mesenchymal stem cells [27]. Knockdown of either α-actinin isoform blunted this stiffness adaptation and significantly reduced cortical stiffness for all ECM stiffnesses. On the most compliant (2 kPa) ECMs, the stiffnesses of α-actinin-1 and α-actinin-4 depleted cells were ∼1.75 kPa and ∼1.5 kPa respectively, representing a ∼40% reduction in stiffness compared to controls. On the stiffest ECMs (>18 kPa), the cortical stiffness of both α-actinin-1 and α-actinin-4 depleted cells achieved a plateau stiffness of ∼2.7 kPa, representing a ∼55% deficiency in cortical stiffness relative to control cells. Taken together, these results demonstrate that each α-actinin isoform plays a critical role in the shape plasticity and adaptation of glioma cells to micromechanical cues, with both isoforms contributing optimally to cell spreading on compliant ECMs and cortical stiffness on stiff ECMs.

### α-Actinin Suppression Increases Vinculin Expression and Recruitment to FAs

**Figure 4:**
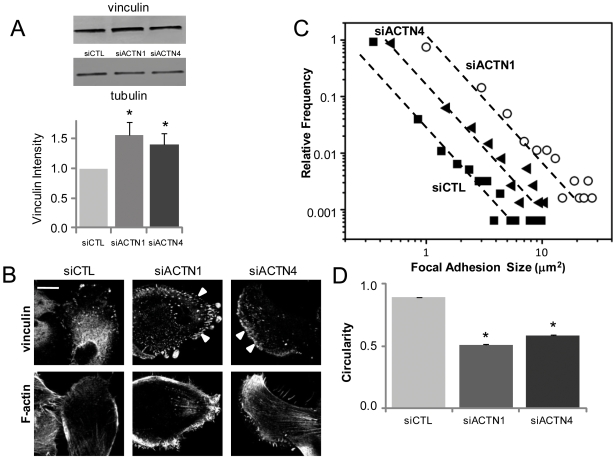
Reciprocal relationship between α-actinin and vinculin expression and recruitment to FAs. (A) Effect of α-actinin isoform suppression on vinculin expression by Western Blot. Vinculin expression is significantly lower (*p<0.05) in both α-actinin-1 and α-actinin-4-depleted cells than in control cells. (B) Effect of α-actinin depletion on vinculin localization. Both α-actinin-1 and α-actinin-4 depleted cells contain more vinculin-positive FAs (arrows) than controls. Scale bar = 20 µm. (C) Quantitative analysis of size and number of vinculin-positive adhesions. The right-shift of the siACTN1 and siACTN4 data relative to control demonstrates that both α-actinin-1 and α-actinin-4 depleted cells possess larger and more numerous adhesions than controls. (D) Circularity of vinculin-positive FAs of siCTL, siACTN1 and siACTN4 cells (*p<0.001). In all cases, data are mean±SEM.

The previous results illustrate that while depletion of either α-actinin isoform significantly reduces cortical stiffness and disrupts actin cytoskeletal architecture, the effects on cell spreading area are surprisingly modest, particularly at high ECM stiffness. To test if this might be explained by compensatory upregulation and redistribution of other FA proteins, we tracked the expression and subcellular localization of vinculin following knockdown of each isoform (Figure 4A, Figures S2 and S6). Depletion of either α-actinin-1 or α-actinin-4 led to enhanced vinculin expression by Western Blot, with an increase of ∼40% in α-actinin-4 knockdown cells, and a more robust increase of ∼55% in α-actinin-1-depleted cells relative to control. Moreover, confocal imaging revealed differences in the number and distribution of vinculin-positive FAs, with α-actinin-1-depleted cells assembling significantly greater numbers of vinculin-positive FAs relative to controls (Figure 4B). Although depletion of α-actinin-4 also enhanced vinculin-positive adhesions relative to control, morphometric analysis revealed that these FAs were smaller and less numerous than in α-actinin-1 depleted cells (Figure 4C). Interestingly, FAs in α-actinin-1-depleted and α-actinin-4-depleted cells were also more elongated than in controls, suggesting that α-actinin depletion yields more mature adhesions (Figure 4D) that also contain pY397FAK (Figure S3).

### Interactions between α-Actinin Isoforms and Activators of Myosin-Based Contractility

The localization of many mechanosensory FA proteins has been shown to specifically depend on activation of myosin motors, which in turn governs cytoskeletal contractility or prestress [28], [29]. To determine if this also holds true for α-actinin-1 and -4, we tracked the localization of these two isoforms in the presence of pharmacologic inhibitors of MLCK, ROCK, and NMMII, all key components of the myosin-based contractility pathway [30], [31], [32]. As expected, Western Blots revealed that treatment of U-373 MG cells with ROCK and MLCK inhibitors markedly reduced levels of MLC phosphorylation relative to either untreated controls or cells in which NMMII ATPase was directly inhibited (Figure S4). While inhibition of MLCK did not lead to dramatic morphological changes (Movie S4), inhibition of either ROCK or NMMII significantly altered cell morphology, with cells exhibiting active membrane ruffles at their leading edges and extending long membrane processes at their trailing edges, consistent with previous observations in other cell systems, including glioma cells (Movie S5) [33], [34].

**Figure 5:**
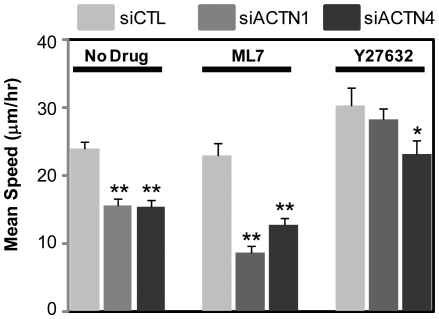
Differential sensitivity of α-actinin-depleted cells to contractile inhibitors. Mean speeds of control, α-actinin-1-depleted, and α-actinin-4-depleted cells for 6 hr following 1 hr of incubation with 10 µM ML7 or 10 µM Y27632. Compared to motility of ML7 treated control cells, knockdown of either isoform significantly reduces cell motility (**p<0.001). Depletion of α-actinin-4 (*p<0.05), but not of α-actinin-1, reduces the motility of Y27632 treated cells to levels observed in untreated cells.

Because both α-actinin isoforms appear to strongly regulate glioma cell motility and glioma cells are sensitive to contractility inhibitors, we reasoned that each contractility inhibitor might differentially oppose or potentiate the reduction in motility observed upon depletion of each isoform. To explore this possibility, we studied the motility of glioma cells under MLCK or ROCK inhibition and in the setting of isoform-specific α-actinin depletion (Figure 5, Movies S6, S7, S8, S9). MLCK inhibition modestly reduced (4%) migration speeds of control cells but did so much more significantly in the setting of either α-actinin-1 (∼60%) or α-actinin-4 (∼40%) suppression. Thus, both α-actinin isoforms act synergistically with MLCK to promote cell motility. In contrast to MLCK, ROCK inhibition enhanced mean migration speeds by ∼25% relative to controls. While this enhancement of cell speed was suppressed slightly but not significantly in α-actinin-1 depleted cells, α-actinin-4 depletion reduced migration speeds to values similar to untreated cells, indicating that the enhancement in cell motility observed with ROCK inhibition may be partially attributed to α-actinin-4.

### Contributions of α-Actinin Isoforms to Myosin Expression and Function

**Figure 6:**
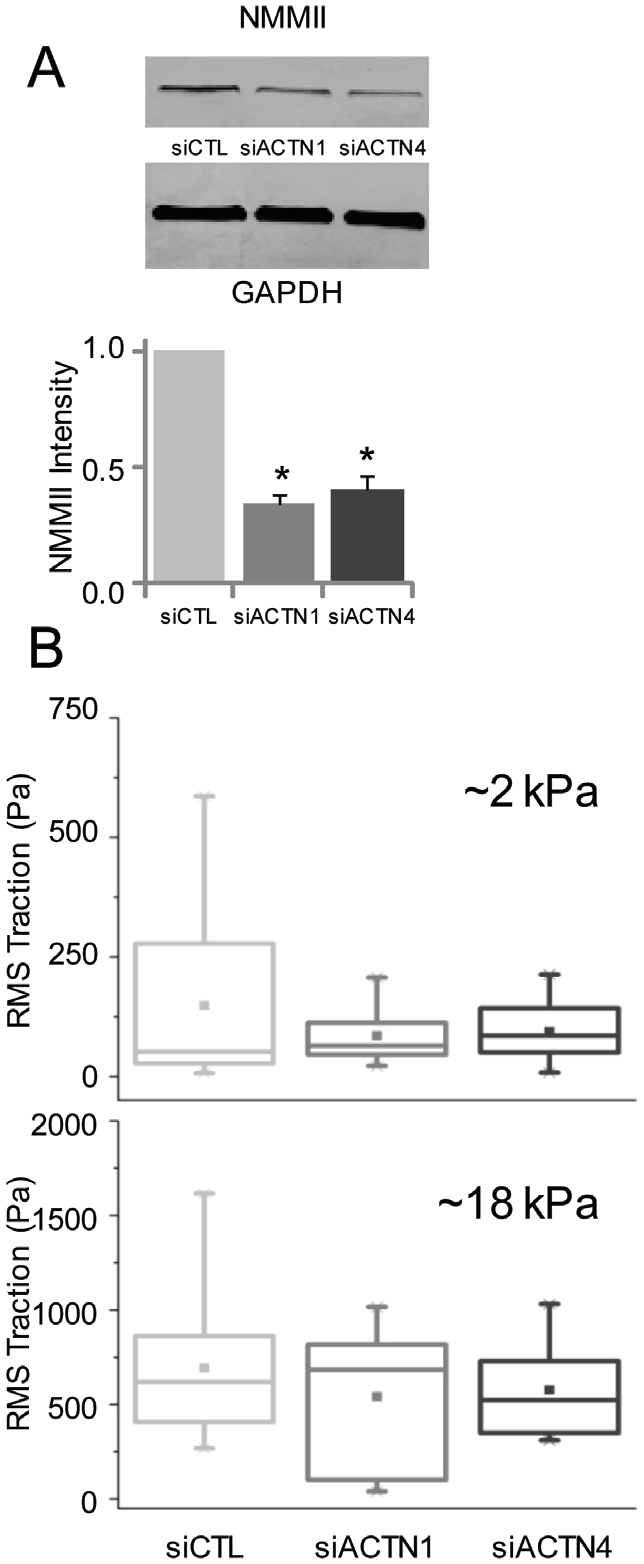
Effect of α-actinin expression on myosin expression and contractility. (A) Effect of α-actinin suppression on NMMII expression by Western Blot. NMMII expression is significantly lower (*p<0.05) in both α-actinin-1 and α-actinin-4-depleted cells than in controls. (B) Effect of α-actinin depletion on traction force generation measured by traction force microscopy. Box-whisker plots of root-mean squared (RMS) traction of siCTL, siACTN1 and siACTN4 cells on 2 kPa and 18 kPa ECM substrates. The error bars mark the maximum and minimum of the data sets, the three horizontal lines mark the 25th, 50th, and 75th percentile of the data, and the point marks the mean. N>10 cells in all cases.

The above results suggest a close association between α-actinin isoforms and NMMII, whose activation is classically associated with increased contractility, increased stiffness, and generation of traction forces [35], [36], [37]. To test whether our observed α-actinin-dependent changes in cellular motility and mechanics were secondary to changes in myosin activity, we compared the levels of NMMII expression and phosphorylation in control and α-actinin-depleted cells (Figure 6A, Figures S5, S6). Indeed, suppression of either α-actinin-1 or α-actinin-4 reduced myosin expression, with a reduction of ∼66% in α-actinin-1 knockdown cells and ∼60% in α-actinin-4 depleted cells relative to control. As expected, suppression of either isoform also led to reduced levels of MLC phosphorylation (pMLC) (Figure S5). To further determine the functional effect of this reduced NMMII expression in α-actinin depleted cells on traction force generation, we used traction force microscopy to compare cell-ECM tractional forces between control and α-actinin-depleted cells on ECMs with elasticites of 2 kPa and 18 kPa, respectively (Figure 6B). This method measures cellular tractional forces (a measure of contractility) by tracking relaxation of ECM-embedded fiduciary markers following cell detachment. On the soft (2 kPa) gels, the mean traction force of control cells, α-actinin-1, and α-actinin-4-depleted cells were 150 Pa, 85 Pa, and 95 Pa, respectively, with knockdown of either subunit eliminating the high-traction (>250 Pa) population observed in control cells (Figure S7). Interestingly, the reduction in mean traction forces closely mirrored the 40% reduction in cell stiffness of actinin-depleted cells observed on 2 kPa gels (Fig. 3). On the stiffer (18 kPa) gels, the mean traction force values shifted to 700 Pa, 540 Pa, and 580 Pa, respectively, and knockdown of either subunit rendered cells unable to generate tractional forces >1.2 kPa. Taken together, these results show that depletion of either α-actinin isoform leads to suppression of NMMII expression and function.

## Discussion

We have examined the contributions of the non-muscle α-actinin isoforms 1 and 4 to the mechanobiology of human glioma cells. We find that these two isoforms localize to different subcellular compartments, and that depletion of either isoform leads to an unexpected increase in vinculin expression and localization to adhesions, which is accompanied by reduced motility. We also show that both isoforms serve mechanosenory functions, with depletion compromising the ability of glioma cells to adapt their structure and mechanics to changes in ECM rigidity. Mechanistic insight into these findings comes from the observation that suppression of either α-actinin isoform reduces expression of NMMII, thereby altering mechanochemical feedback between tumor cells and the ECM and reducing maximal traction forces. Taken together, our data support a model in which α-actinin isoforms participate in mechanochemical feedback between glioma cells and the ECM, with expression and localization of these molecules regulating NMMII-dependent processes, including adhesion, motility, stiffening, and contractility. While these results are clear, two points are worth noting: First, the detailed mechanisms that link suppression of α-actinin to altered cell migration and mechanics remain to be elucidated, and it is likely that other elements of the adhesive and contractile machinery besides NMMII and vinculin play important roles. Second, we chose not to attempt to rescue effects of α-actinin-depletion by overexpressing exogenous α-actinin, both because low double-transfection efficiencies make obtaining meaningful statistics from single-cell measurements challenging, and because α-actinin overexpression has previously been observed to phenocopy α-actinin depletion (e.g., with respect to cell migration, as described in the introduction). Thus, we cannot completely rule out the possibility that some of our observed effects may be due in part to secondary effects of α-actinin depletion.

Our findings may bear implications for GBM invasion in vivo. The direct role of NMMII in GBM invasiveness was first demonstrated by studies in which treatment with MLCK inhibitors completely abrogated glioma motility [6]. Three isoforms of NMMII are known to exist (NMMIIA, B, C); NMMIIA is upregulated in human glioma xenografts, and both NMMIIA and NMMIIB are required to squeeze through pores smaller than the size of nuclei [38]. Our studies reveal a causal connection between α-actinin and NMMII in which depletion of either α-actinin isoform reduces NMMII expression. This close interplay between α-actinin and NMMII in modulating contractility is consistent with biophysical studies of gels composed of F-actin, NMMII and α-actinin, which have found that bulk contractility of the gel occurs only above a threshold α-actinin concentration [39]. The actin-crosslinking activity of NMMII in the initial stages of adhesion has been demonstrated in recent studies where overexpression of wild-type NMMII in α-actinin-depleted CHO cells was able to restore adhesion maturation [40]. In human aortic endothelial cells, the increase in cell prestress obtained with knockdown of α-actinin-1 suggests that α-actinin depletion increases actomyosin interactions and raises the possibility that α-actinin and NMMII compete for common binding sites in crosslinking F-actin [41]. The cell softening observed in our studies upon α-actinin knockdown is also consistent with a published report in which microinjection of α-actinin not only increased the stiffness of 3T3 fibroblasts but also the degree of mechanical heterogeneity of the cytoplasm, as measured by particle tracking microrheology [42].

Cell migration requires the coordinated extension of membrane protrusions, formation of transient adhesions along the membrane extensions, and NMMII-dependent detachment of the cell rear [43]. The activity of NMMII is controlled by MLC phosphorylation, and recent efforts to elucidate regional variations in MLC phosphoregulation have suggested that MLCK preferentially regulates dynamic adhesions at the cell periphery and that ROCK regulates more stable FAs at the cell center [44], [45]. We find that motility is reduced most dramatically by suppressing those isoforms and inhibiting MLCK. Because the rapid turnover of FAs at the leading edge needed for migration depends on MLC phosphorylation, we hypothesize that MLCK utilizes both α-actinin isoforms in order to promote motility. Similarly, because the enhancement in motility observed upon ROCK inhibition is reduced by α-actinin-4 depletion, we suspect that this enhancement may require mobilization of α-actinin-4. Potential mechanisms for this effect might include increased interaction between α-actinin-4 and pMLC, which translocates to membrane protrusions in a Rac-dependent manner [46].

The adaptation of glioma cells to changes in ECM stiffness observed here is reminiscent of the finding that cultured fibroblasts match their stiffness to the underlying ECM substrate for ECM stiffnesses up to 5 kPa and reach a maximal stiffness on ECM substrates [26]. The failure of α-actinin-depleted cells to exhibit this stiffness adaptation may be due to the requirement for α-actinin to crosslink cortical actin and stabilize connections between F-actin and β integrin subunits in adhesions, which would be expected to require strongest reinforcement on high-rigidity ECMs, which support large traction forces [47]. Conversely, both α-actinin isoforms contribute most strongly to spreading area at *low* ECM rigidity. We speculate that this may be due to the compensatory effects of vinculin, whose expression and localization to FAs significantly increase on rigid ECMs in the setting of α-actinin suppression, thereby preserving cell shape but not necessarily cortical stiffness. This concept is supported by previous studies in which vinculin suppression reduced spreading, stress fiber formation, FA formation, and lamellipodial extension [48]. This compensatory effect may itself depend on ECM rigidity, such that α-actinin-depleted cells cultured on compliant ECMs are much less capable of recruiting additional vinculin to FAs than their counterparts on rigid ECMs. As a result, we observe negligible deficiencies in cell spreading of α-actinin depleted cells on stiff substrates but see these differences amplify as matrix stiffness is reduced. Enhanced expression of α-actinin may switch GBM tumor cells from a strongly adhesive and low-motility phenotype to a highly contractile and invasive phenotype, and the possibility that invading glioma cells may stiffen the brain parenchyma as they invade [7] suggests a complex interplay between ECM rigidity, α-actinin, and vinculin.

One particularly unexpected finding from these studies is that α-actinin-depleted cells spread extensively on stiff ECMs despite having relatively low NMMII expression and low cellular stiffness and prestress. While this result may be inconsistent with the conventional notion that high cellular traction is always needed to support spreading, it is not without precedent. For example, Janmey and coworkers recently reported that under some conditions, filamin A-null cells increase their spreading in response to increasing ECM stiffness while maintaining a relatively constant cell stiffness [49], indicating that stiffness and spreading are not strictly coupled. Similarly, we recently showed that when U-373 MG cells cultured on highly compliant ECMs are treated with ROCK or NMMII inhibitors, which presumably reduce contractility, they paradoxically begin to spread and migrate [7]. These and other studies suggest the possibility that specific actin crosslinking proteins may modulate the degree of coupling between cell spreading and cellular contractility.

In conclusion, we have investigated the contributions of α-actinin-1 and α-actinin-4 to mechanobiological behaviors of human glioma cells and shown that suppressing each isoform bears significant but distinct implications for tumor cell morphology, motility, mechanics, rigidity-sensing, and force generation. As described earlier, expression of α-actinin has been strongly correlated with invasive behavior of tumor cells in GBM and other cancers, although the roles of each isoform remain unclear. Our findings raise the exciting possibility that enhanced expression of α-actinin isoforms in tumors could speed motility *in vivo* by allowing increased NMMII-generated tractional forces against the ECM or altering processing of microenvironmental mechanical cues. Since our level of actinin suppression is partial, some of our observations may be consequences of altered myosin or vinculin expression. However, it is also possible that modulation of α-actinin expression in vivo may trigger compensatory changes in the expression and function of other FA proteins, as we observe in culture. Testing this hypothesis will require asking whether normalizing α-actinin overexpression can reduce invasive behavior *in vivo*. Given the importance of NMMII to navigating microstructural barriers in 3D ECMs, it will also be important to revisit these behaviors in the context of 3D ECMs that present more complex combinations of mechanical and topological cues.

## Supporting Information

Figure S1 Immunofluorescence co-localization of pY397FAK with (A) α-actinin-1 and (B) α-actinin-4 following siRNA-mediated suppression of each α-actinin isoform in U-373 MG cells.In both (A) and (B), the left, middle, and right columns show results for control, α-actinin-1 and α-actinin-4-directed siRNAs, respectively. In each case, the top, middle, and bottom rows show immunolocalization of the relevant α-actinin isoform (green), pY397FAK (red), and the merged signal, respectively. Scale Bar = 20 µm.
(6.13 MB TIF)

Figure S2 Immunofluorescence localization of vinculin (red) in control cells (siCTL) (left column), α-actinin-1-depleted cells (siACTN1) (middle column), and α-actinin-4-depleted cells (siACTN4). Five representative images of each category are shown. Scale Bar = 20 µm.
(7.44 MB TIF)

Figure S3 Immunofluorescence localization of pY397FAK in control cells (siCTL) (left column) α-actinin-1-depleted cells (siACTN1) (middle column) and α-actinin-4-depleted cells (siACTN4) (right column). Cells have been co-stained for pY397FAK (red) and F-actin (green). Scale Bar = 20 µm.
(7.36 MB TIF)

Figure S4 Effect of nonmuscle myosin II pathway inhibitors on phosphorylated MLC (pMLC) levels in U-373 MG cells by Western Blot. From left to right, the lanes represent lysates from untreated controls (CTL), cells treated with 10 µM ML7 (ML7), cells treated with 10 µM Y27632 (Y27), and cells treated with 10 µM blebbistatin (Blebb). The bottom bands are corresponding GAPDH loading controls.M ML7 (ML7), cells treated with 10 µM Y27632 (Y27), and cells treated with 10 µM blebbistatin (Bleb). The bottom bands are corresponding GAPDH loading controls.
(1.10 MB TIF)

Figure S5 Effect of α-actinin suppression on pMLC levels by Western Blot. From left to right, the lanes represent lysates from cells treated with control siRNA (siCTL), siRNA against α-actinin-1 (siACTN1), and siRNA against α-actinin-4 (siACTN4). The bottom bands are corresponding GAPDH loading controls.
(1.03 MB TIF)

Figure S6 Time course of effect of α-actinin-1 suppression on expression of vinculin and NMMII by Western Blot. The leftmost lane represents lysates from cells treated with control siRNA for 8 hours (siCTL). Subsequent lanes represent cells treated with siRNA against α-actinin-1 (siACTN1) for 8, 16, and 24 hours, respectively. Blots reveal a time-dependent suppression in NMMII expression (top bands) and a gradual increase in vinculin expression (middle bands). The bottom bands represent the GAPDH loading controls.
(1.18 MB TIF)

Figure S7 Traction maps exerted by siCTL, siACTN1, and siACTN4 cells on 2 kPa gels. Traction fields have been computed using Fourier Transform Traction Cytometry. Arrows show the direction and relative magnitude of traction forces. Color code shows the magnitude of traction forces in Pa. Note the differences in scales.
(6.66 MB TIF)

Movie S1 Phase contrast imaging of U373-MG cells transfected with control siRNA (siCTL) migrating on collagen-coated tissue culture plastic over a period of at least 6 hours. Frames were acquired every 15 min.
(9.05 MB AVI)

Movie S2 Phase contrast imaging of U373-MG cells transfected with α-actinin-1 siRNA (siACTN1) migrating on collagen-coated tissue culture plastic over a period of at least 6 hours. Frames were acquired every 15 min.
(9.05 MB AVI)

Movie S3 Phase contrast imaging of U373-MG cells transfected with α-actinin-4 siRNA (siACTN4) migrating on collagen-coated tissue culture plastic over a period of at least 6 hours. Frames were acquired every 15 min.
(9.05 MB AVI)

Movie S4 Phase contrast imaging of U373-MG cells transfected with control siRNA (siCTL) and exposed to 10 µM ML7, migrating on collagen-coated tissue culture plastic over a period of at least 6 hours. Frames were acquired every 15 min.
(10.14 MB AVI)

Movie S5 Phase contrast imaging of U373-MG cells transfected with control siRNA (siCTL) and exposed to 10 µM Y27632, migrating on collagen-coated tissue culture plastic over a period of at least 6 hours. Frames were acquired every 15 min.
(10.14 MB AVI)

Movie S6 Phase contrast imaging of U373-MG cells transfected with α-actinin-1 siRNA (siACTN1) and exposed to 10 µM ML7, migrating on collagen-coated tissue culture plastic over a period of at least 6 hours. Frames were acquired every 15 min.
(10.14 MB AVI)

Movie S7 Phase contrast imaging of U373-MG cells transfected with α-actinin-1 siRNA (siACTN1) and exposed to 10 µM Y27632, migrating on collagen-coated tissue culture plastic over a period of at least 6 hours. Frames were acquired every 15 min.
(10.14 MB AVI)

Movie S8 Phase contrast imaging of U373-MG cells transfected with α-actinin-4 siRNA (siACTN4) and exposed to 10 µM ML7, migrating on collagen-coated tissue culture plastic over a period of at least 6 hours. Frames were acquired every 15 min.
(10.14 MB AVI)

Movie S9 Phase contrast imaging of U373-MG cells transfected with α-actinin-4 siRNA (siACTN4) and exposed to 10 µM Y27632, migrating on collagen-coated tissue culture plastic over a period of at least 6 hours. Frames were acquired every 15 min.
(10.14 MB AVI)
