# Supplementary figures and images for: Isoform-Specific Contributions of α-Actinin to Glioma Cell Mechanobiology
Source: PLoS One. 2009 Dec 23;4(12):e8427. doi: 10.1371/journal.pone.0008427 (PMC2793025; doi:10.1371/journal.pone.0008427)

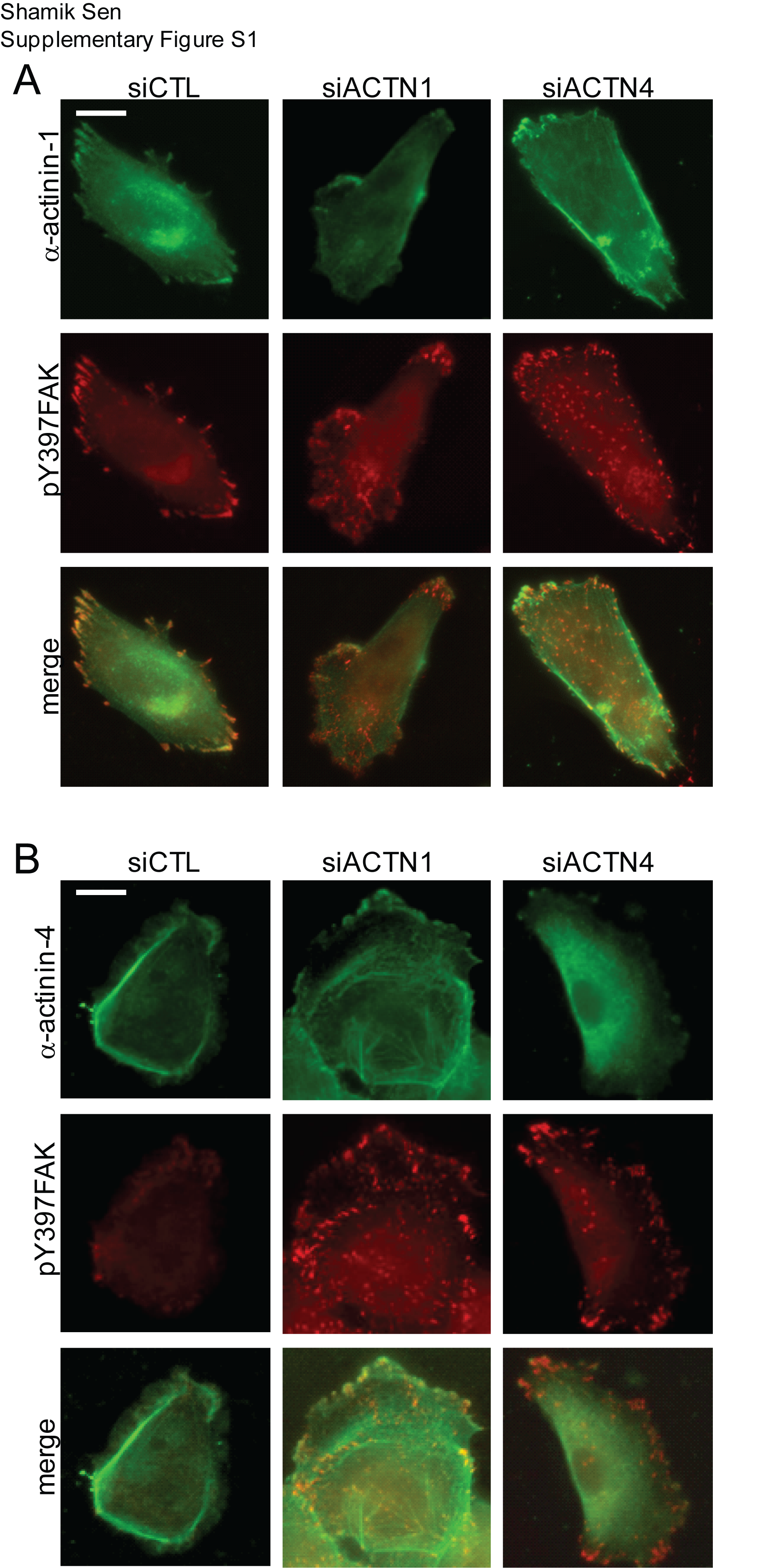

Supplement: Figure S1 — Immunofluorescence co-localization of pY397FAK with (A) α-actinin-1 and (B) α-actinin-4 following siRNA-mediated suppression of each α-actinin isoform in U-373 MG cells.In both (A) and (B), the left, middle, and right columns show results for control, α-actinin-1 and α-actinin-4-directed siRNAs, respectively. In each case, the top, middle, and bottom rows show immunolocalization of the relevant α-actinin isoform (green), pY397FAK (red), and the merged signal, respectively. Scale Bar = 20 µm. (6.13 MB TIF) [file pone.0008427.s001.tif]

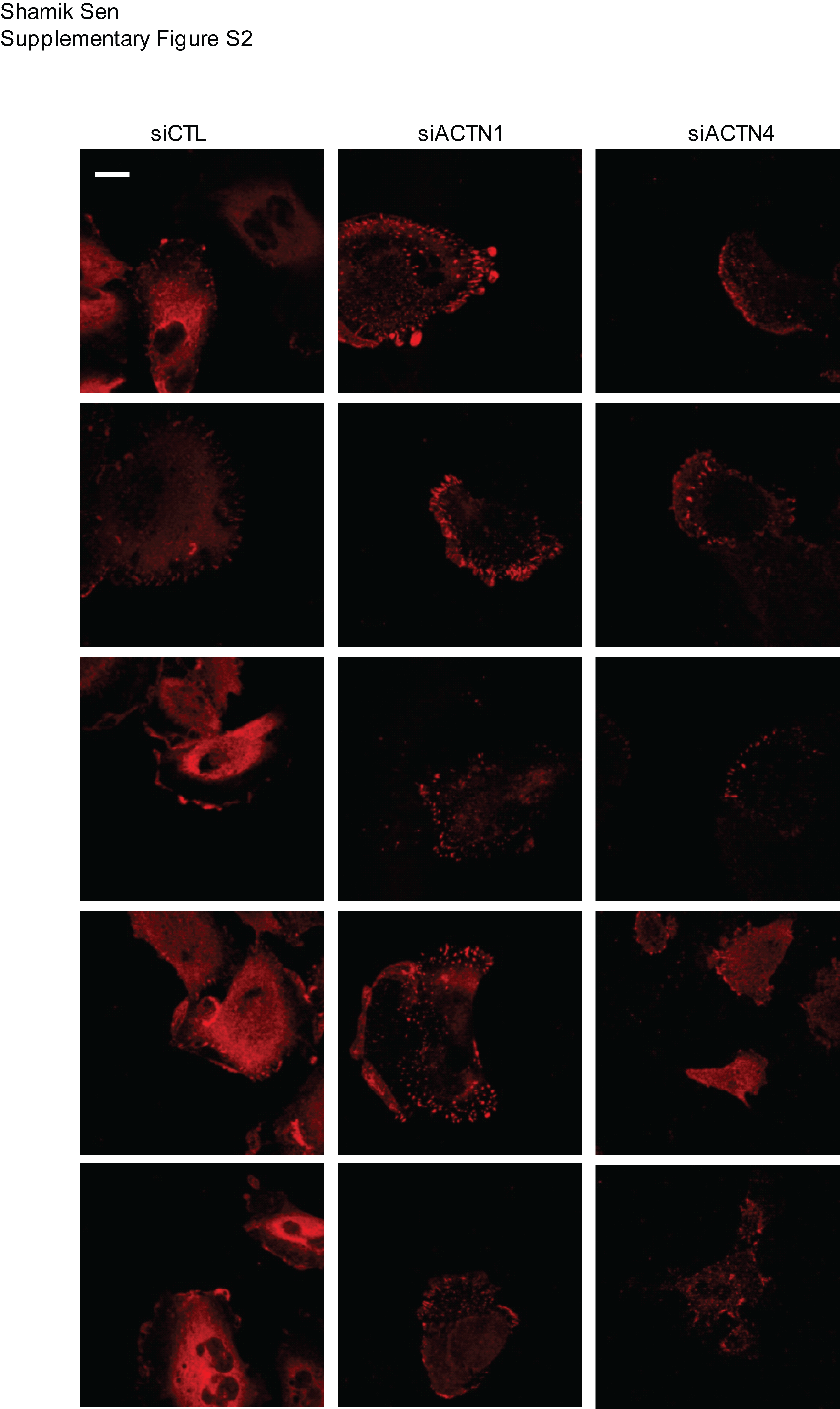

Supplement: Figure S2 — Immunofluorescence localization of vinculin (red) in control cells (siCTL) (left column), α-actinin-1-depleted cells (siACTN1) (middle column), and α-actinin-4-depleted cells (siACTN4). Five representative images of each category are shown. Scale Bar = 20 µm. (7.44 MB TIF) [file pone.0008427.s002.tif]

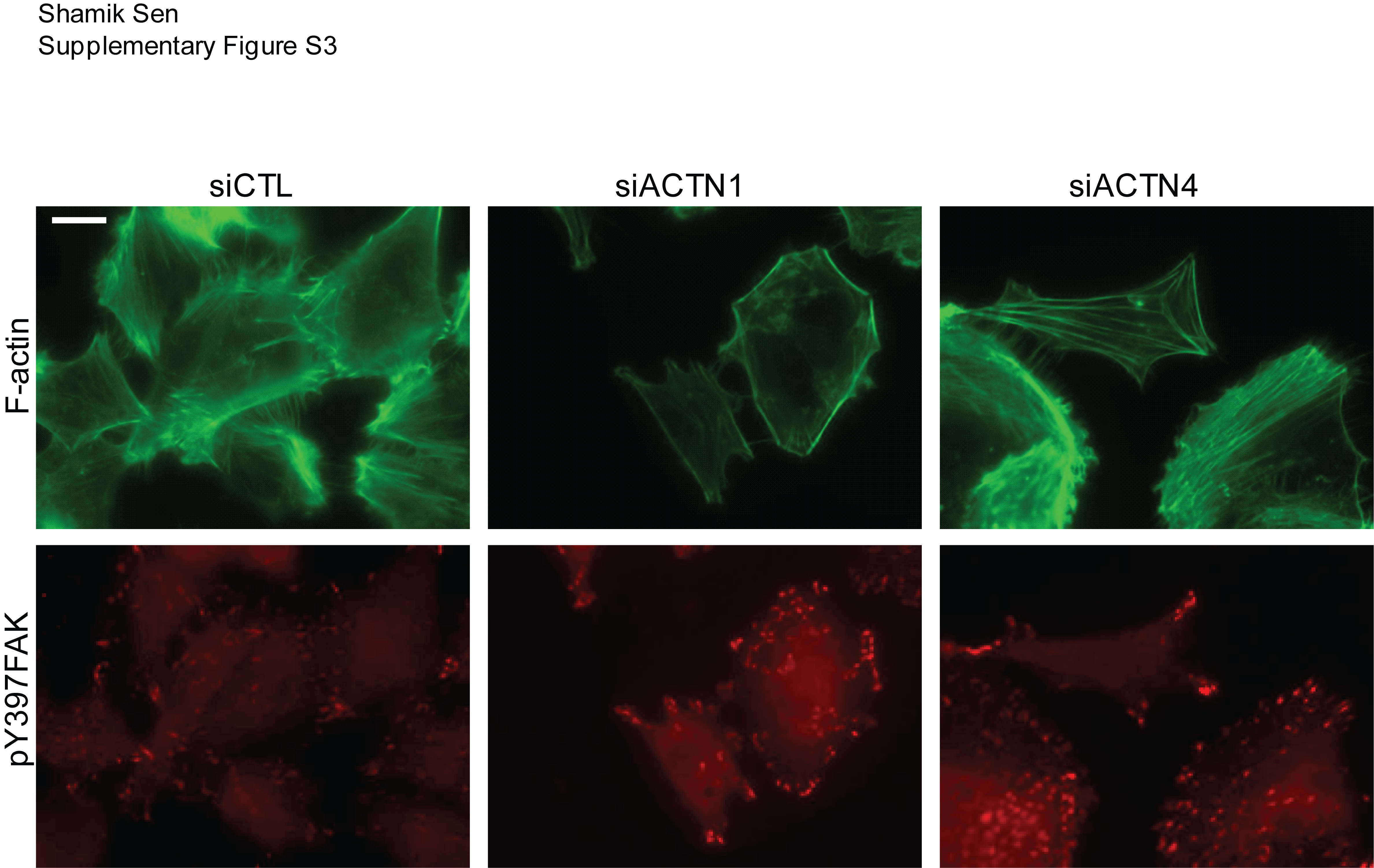

Supplement: Figure S3 — Immunofluorescence localization of pY397FAK in control cells (siCTL) (left column) α-actinin-1-depleted cells (siACTN1) (middle column) and α-actinin-4-depleted cells (siACTN4) (right column). Cells have been co-stained for pY397FAK (red) and F-actin (green). Scale Bar = 20 µm. (7.36 MB TIF) [file pone.0008427.s003.tif]

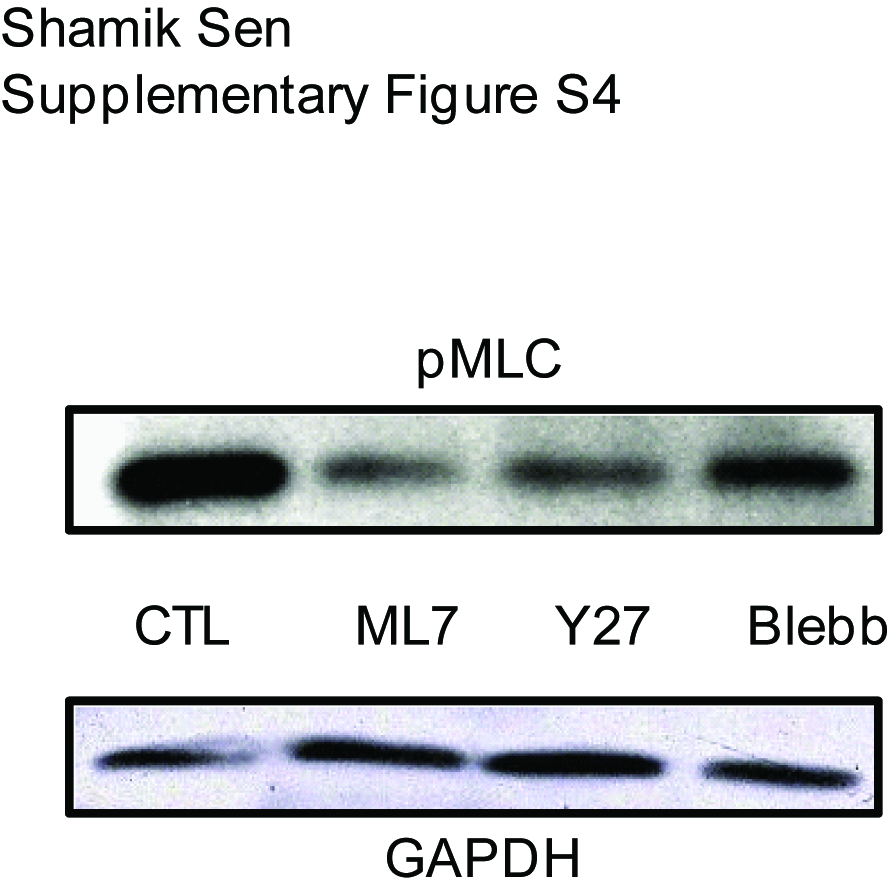

Supplement: Figure S4 — Effect of nonmuscle myosin II pathway inhibitors on phosphorylated MLC (pMLC) levels in U-373 MG cells by Western Blot. From left to right, the lanes represent lysates from untreated controls (CTL), cells treated with 10 µM ML7 (ML7), cells treated with 10 µM Y27632 (Y27), and cells treated with 10 µM blebbistatin (Blebb). The bottom bands are corresponding GAPDH loading controls.M ML7 (ML7), cells treated with 10 µM Y27632 (Y27), and cells treated with 10 µM blebbistatin (Bleb). The bottom bands are corresponding GAPDH loading controls. (1.10 MB TIF) [file pone.0008427.s004.tif]

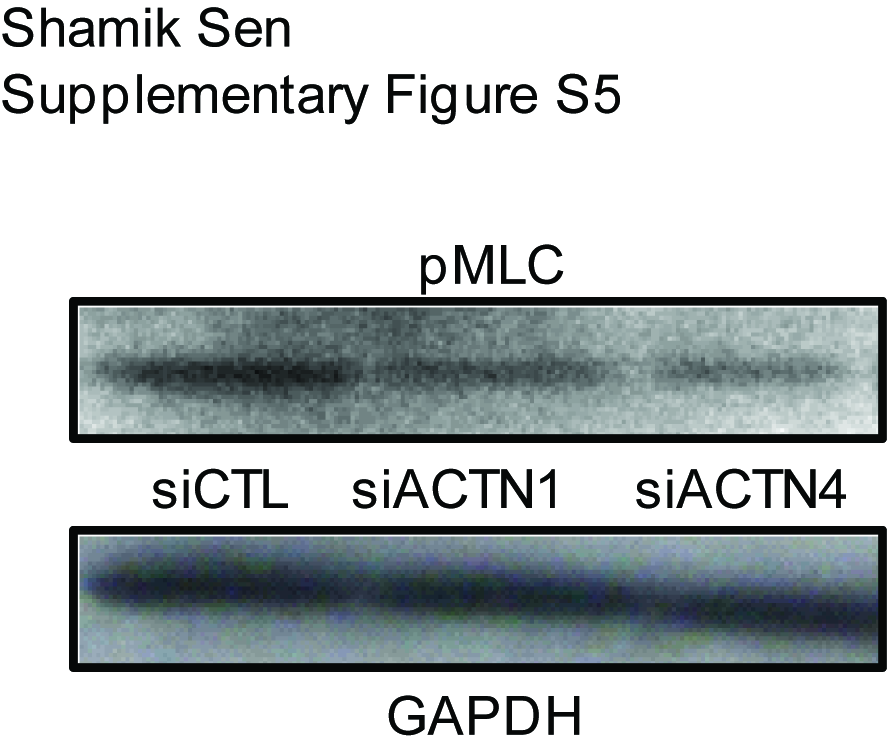

Supplement: Figure S5 — Effect of α-actinin suppression on pMLC levels by Western Blot. From left to right, the lanes represent lysates from cells treated with control siRNA (siCTL), siRNA against α-actinin-1 (siACTN1), and siRNA against α-actinin-4 (siACTN4). The bottom bands are corresponding GAPDH loading controls. (1.03 MB TIF) [file pone.0008427.s005.tif]

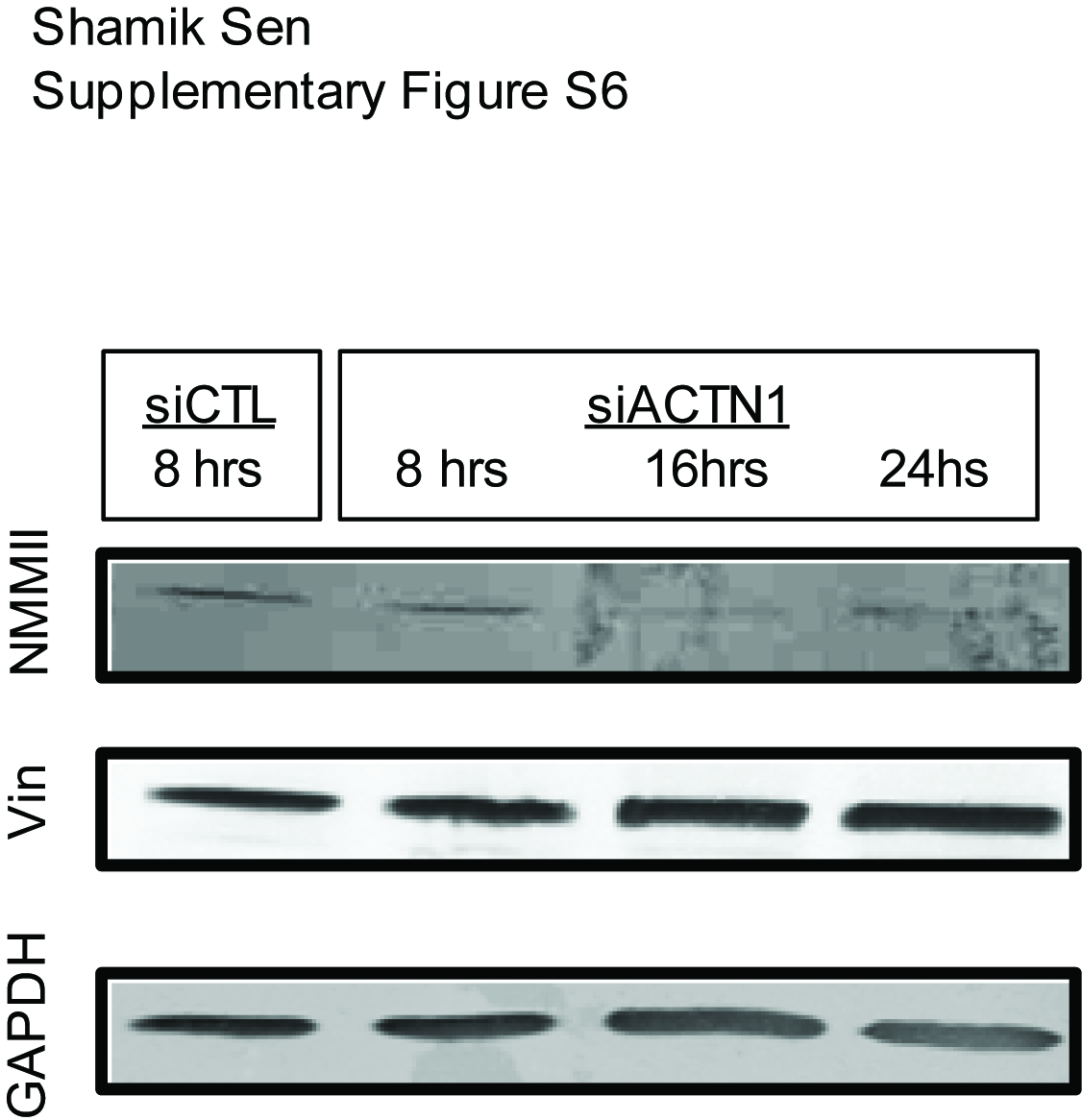

Supplement: Figure S6 — Time course of effect of α-actinin-1 suppression on expression of vinculin and NMMII by Western Blot. The leftmost lane represents lysates from cells treated with control siRNA for 8 hours (siCTL). Subsequent lanes represent cells treated with siRNA against α-actinin-1 (siACTN1) for 8, 16, and 24 hours, respectively. Blots reveal a time-dependent suppression in NMMII expression (top bands) and a gradual increase in vinculin expression (middle bands). The bottom bands represent the GAPDH loading controls. (1.18 MB TIF) [file pone.0008427.s006.tif]

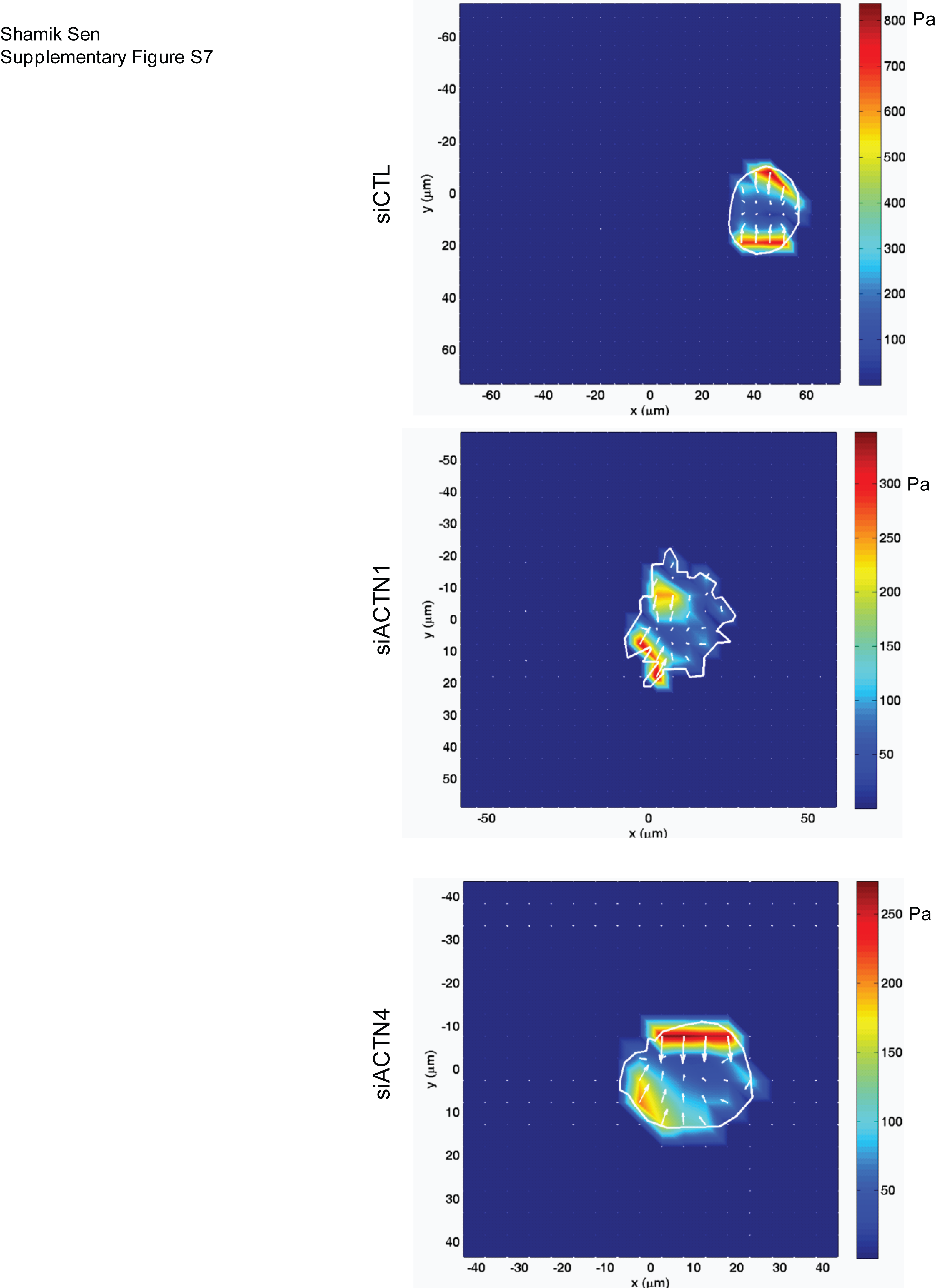

Supplement: Figure S7 — Traction maps exerted by siCTL, siACTN1, and siACTN4 cells on 2 kPa gels. Traction fields have been computed using Fourier Transform Traction Cytometry. Arrows show the direction and relative magnitude of traction forces. Color code shows the magnitude of traction forces in Pa. Note the differences in scales. (6.66 MB TIF) [file pone.0008427.s007.tif]
